# Identification and Functional Characterization of *IDS* Gene Mutations Underlying Taiwanese Hunter Syndrome (Mucopolysaccharidosis Type II)

**DOI:** 10.3390/ijms21010114

**Published:** 2019-12-23

**Authors:** Hsiang-Yu Lin, Ru-Yi Tu, Schu-Rern Chern, Yun-Ting Lo, Sisca Fran, Fang-Jie Wei, Sung-Fa Huang, Shin-Yu Tsai, Ya-Hui Chang, Chung-Lin Lee, Shuan-Pei Lin, Chih-Kuang Chuang

**Affiliations:** 1 Department of Medical Research, MacKay Memorial Hospital, New Taipei City 25160, Taiwan; lxc46199@ms37.hinet.net (H.-Y.L.); likemaruko@hotmail.com (R.-Y.T.); srchern@mmh.org.tw (S.-R.C.); fransiscazen@gmail.com (S.F.); jessicacat83@gmail.com (F.-J.W.); 2 Department of Pediatrics, MacKay Memorial Hospital, Taipei 10449, Taiwan; 3 MacKay Junior College of Medicine, Nursing and Management, New Taipei City 25245, Taiwan; 4 Department of Medicine, MacKay Medical College, New Taipei City 25245, Taiwan; 5 Department of Medical Research, China Medical University Hospital, China Medical University, Taichung 40402, Taiwan; 6 Rare Disease Center, MacKay Memorial Hospital, Taipei 10449, Taiwan; andy11tw.e347@mmh.org.tw (Y.-T.L.); cindyyayahappy@gmail.com (S.-Y.T.); wish1001026@gmail.com (Y.-H.C.); 7 Department of Laboratory Medicine, MacKay Memorial Hospital, New Taipei City 25160, Taiwan; alpha-67@yahoo.com.tw; 8 Department of Pediatrics, MacKay Memorial Hospital, Hsinchu 30071, Taiwan; clampcage@gmail.com; 9 Institute of Clinical Medicine, National Yang-Ming University, Taipei 11221, Taiwan; 10 Department of Infant and Child Care, National Taipei University of Nursing and Health Sciences, Taipei 11219, Taiwan; 11 College of Medicine, Fu-Jen Catholic University, New Taipei City 24205, Taiwan

**Keywords:** Hunter syndrome, mucopolysaccharidosis II (MPS II), iduronate-2-sulfatase (IDS) gene, COS-7 cell transfection, IDS enzyme activity, genotype–phenotype correlation.

## Abstract

Hunter syndrome (mucopolysaccharidosis II; MPS II) is caused by a defect of the iduronate-2-sulfatase (*IDS*) gene. Few studies have reported integrated mutation data of Taiwanese MPS II phenotypes. In this study, we summarized genotype and phenotype correlations of confirmed MPS II patients and asymptomatic MPS II infants in Taiwan. Regular polymerase chain reaction and DNA sequencing were used to identify genetic abnormalities of 191 cases, including 51 unrelated patients with confirmed MPS II and 140 asymptomatic infants. *IDS* activity was analyzed in individual novel *IDS* variants using in vitro expression studies. Nineteen novel mutations were identified, in which the percentages of IDS activity of the novel missense mutations c.137A>C, c.311A>T, c.454A>C, c.797C>G, c.817C>T, c.998C>T, c.1106C>G, c.1400C>T, c.1402C>T, and c.1403G>A were significantly decreased (*p* < 0.001), c.254C>T and c.1025A>G were moderately decreased (*p* < 0.01), and c.851C>T was slightly decreased (*p* < 0.05) comparing with normal enzyme activity. The activities of the other six missense mutations were reduced but were insignificant. The results of genomic studies and their phenotypes were highly correlated. A greater understanding of the positive correlations may help to prevent the irreversible manifestations of Hunter syndrome, particularly in infants suspected of having asymptomatic MPS II. In addition, urinary glycosaminoglycan assay is important to diagnose Hunter syndrome since gene mutations are not definitive (could be non-pathogenic).

## 1. Introduction

Hunter syndrome (mucopolysaccharidosis II; MPS II) (OMIM 309900) is an X-linked recessive lysosomal storage disorder (LSD). MPS II is caused by a deficiency in iduronate-2-sulfatase activity (*IDS* gene; Hunter: OMIM#309900; EC 3.1.6.13), which is involved in the lysosomal degradation of heparan sulfate (HS) and dermatan sulfate (DS). There are two major clinical forms, including mild and severe forms based on age at onset and severity of clinical manifestations [1,2,3,4]. The heterogeneity of the syndrome is assumed to reflect different mutations at the *IDS* locus affecting the expression of the IDS protein (enzyme), stability, and catalyzation function. The severe form of MPS II is characteristic by early somatic abnormalities together with skeletal deformities, hepatosplenomegaly, and progressive cardiopulmonary deterioration. In the severe form of MPS II, neurological damage presents progressively and prominently as developmental delay and intellectual disability, often concomitant with neurodegeneration; whereas patients with the mild form of MPS II usually have the attenuated somatic complications without mental disability. MPS II is the most common type of MPS, accounting for 52% of all diagnosed MPS cases in Taiwan, and the prevalence in northeast Asia is very similar [5,6,7]. The birth prevalence of MPS II in different populations was varied, i.e., 1.07 per 100,000 live births in Taiwan [7,8]; 0.84 per 100,000 live births in Japan; 0.74 per 100,000 live births in South Korea; 0.29 per 100,000 live births in the United States; 0.27, 1.09, and 0.67 per 100,000 live births in Denmark, Portugal, and The Netherlands, respectively [8]. However, integrated mutation data of the Taiwanese MPS II phenotype are currently lacking.

The human *IDS* gene has been mapped to chromosome Xq28.1, spans approximately 24 kb, and contains 9 exons [9]. The whole *IDS* gene has been sequenced, and an *IDS*-like pseudogene, comprising copies of exons 2 and 3 and intron 7, has been located about 20 kb from the active gene [10,11,12]. The full-length cDNA is 1650 bp encoding a 550 amino-acid polypeptide, which has shown a high degree of homology with the sulfatase protein family [13]. To date, more than 541 different mutations underlying MPS II have been identified [www.HGMD.cf.ac.uk/]. The frequency of large alterations (complete or partial gene deletions and large rearrangements) is about 28.2%, however, the majority of the identified mutations (71.8%) are small deletions, insertions, or single base substitutions (missense mutations, nonsense mutations, and mutations affecting splicing). Haplotype analysis has shown that mutations occur more frequently in male meiosis. Even though the disease is almost reported in males, rare sporadic cases in females do occur. Affected females generally have low levels of IDS activity associated with a mild clinical phenotype. The result of either an X chromosome anomaly or homozygosity for the mutated gene, but most frequently, is the consequence of skewed X chromosome inactivation [14,15].

Accurate knowledge of the specific mutations involved in this disease may help to clarify the relationships between genotype and phenotype in individual patients and allow for the identification of female carriers. To characterize the biochemical and molecular defects in IDS-deficient patients and their families, this study was designed to identify *IDS* gene mutations in a group of Taiwanese patients with MPS II. We analyzed the genotype–phenotype relationships in all 51 *IDS* variants currently known in Taiwan using integrated clinical data with in vitro expression studies and a mass spectrometry-based assay of urinary glycosaminoglycans (uGAGs) to investigate and predict the likelihood of novel *IDS* variants for the severe or attenuated phenotype.

## 2. Results

### 2.1. Mutations of the IDS Gene by Sequencing Analysis

**Figure 1:**
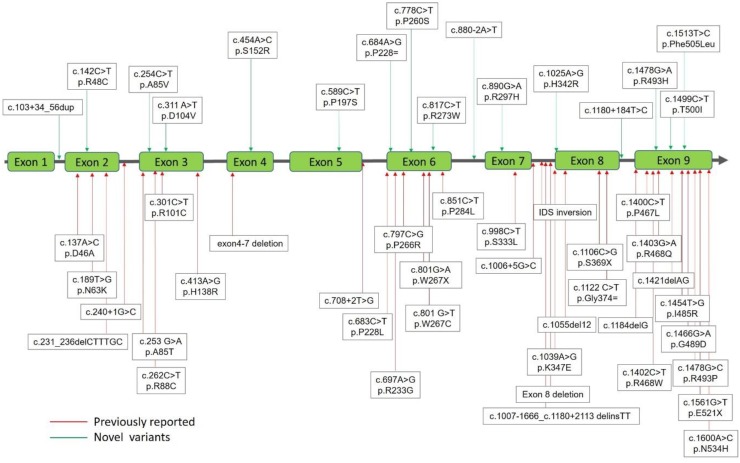
Mutations of the iduronate-2-sulfatase (IDS) gene underlying Taiwanese Hunter syndrome. A total of 51 mutations of the *IDS* gene underlying Taiwanese Hunter syndrome were found, in those 35 have been reported previously (red lines with arrows) and the other 16 were novel mutations (green lines with arrows).

Sequencing data were obtained by scanning through the data to identify anomalies using Applied Biosystems Sequence Scanner Software v2.0 Sequence Trace Viewer and Editor (Applied Biosystems Co., CA, USA) and manual progressive alignment. A total of 51 mutations of the *IDS* gene from the 191 cases, including the confirmed patients (*n* = 51) and infants suspected of having MPS II (*n* = 140) that were identified. The suspected MPS II infants were classified into two groups: Those with either 1) “positive” uGAG biochemistry examinations, reduction in leukocyte IDS enzyme activity, and identified IDS variations (*n* = 7); or 2) “negative” uGAG biochemistry examinations, reduction in leukocyte IDS enzyme activity and identified *IDS* variations (*n* = 133). The 51 mutations of the *IDS* gene included 32 missense (62.7%), 3 nonsense (5.9%), 2 silent (3.9%), 6 splicing (11.8%), 4 small deletions (7.8%), 3 gross deletions (5.9%), and 1 complex inversion (2%) (Table 1). Of these mutations, 35 were reported and verified as being pathogenic for MPS II with varying degrees of severity or non-pathogenic [13,14,15,16,17,18,19,20,21,22,23,24,25,26,27,28,29,30,31,32,33,34,35,36], and the other 16 were novel mutations (Figure 1), which needed to be verified according to individual IDS activity by using in vitro expression studies.

The 32 missense mutations are listed in Table 1. Most of the missense variations were verified as being pathogenic genes for Hunter syndrome [16,17,18,19,20,21,22,23,24,25,26,27,28,29,30,31,32,33,34,35,36,37,38,39], except for 12 novel mutations, i.e., c.142C>T (p.R48C), c.254C>T (p.A85V), c.311A>T (p.D104V), c.454A>C (p.S152R), c.589C>T (p.P197S), c.778C>T (p.P260S), c.817C>T (p.R273W), c.890G>A (p.R297H), c.1025A>G (p.H342R), c.1478G>A (p.R493H), c.1499C>T (p.T500I), and c.1513T>C (p.F505L). Most of these missense mutations have been identified by the NBS program for MPS in Taiwan since August 2015. The phenotypes of the confirmed MPS II patients varied from the attenuated (mild form) to the severe form according to the IQ test, DQ tests, and WeeFIM questionnaire scores. The phenotypes of missense mutations (14/32) from the NBS program were uncertain due to the patients being asymptomatic during the infantile period, and the sequence variant classification, according to the American College of Medical Genetics (ACMG) criteria, were also demonstrated in Table 1. The ACMG Standards and Guidelines can provide an interpretation for evaluating evidence for sequence variants observed in patients with suspected MPS II (primarily Mendelian) [40]. Two of the missense mutations (c.301C>T, p.R101C and c.851C>T, p.P284L) have been reported to be non-pathogenic *IDS* mutations and are regarded as being the attenuated form [21,24]. In the current study, 6 infants had the c.301C>T mutation in which the construct expressed a high activity of about 97% ± 9% of the wild-type activity reported by Keeratichamroen et al. [18]. The predictive ability for the onset of MPS signs or symptoms for infants with this variant may be extraordinarily low or even unremarkable, as suggested by our analysis showing normal leukocyte IDS activity, ranging from 7.74 to 43.9 (25.82 ± 9.04) μmol/g protein/4 h, and negative uGAG tests, particularly the quantities of DS and HS (less than the cut-off values, <0.80 μg/mL for DS and < 0.78 μg/mL for HS). The diagnosis in these cases could be because the mutation was non-pathogenic and may or may not cause MPS signs or symptoms, however, further studies are needed to clarify this issue. Kosuga et al. previously verified that the disease phenotype of the missense mutation c.851C>T (p.P284L) is the attenuated form [24]. Another missense mutation c.1400C>T (p.P467L) has been reported to be pathogenic to cause severe MPS manifestations [27,28]. The infant and another highly suspected infant with the novel missense mutation c.311A>T (p.D104V) who did not have pre-symptoms received Enzyme Replacement Therapy (ERT) (idursulfase) or ERT plus hematopoietic stem cell transplantation (HSCT) at the ages of 1.11 and 0.55 years old, respectively, due to definite evidence of a family history.

The nonsense mutations, c.801G>A (W267X) and c.1561G>T (E521X), have been reported previously [17], and showed the severe phenotype due to protein alteration in Trp-Stop and Glu-Stop, respectively. Another nucleotide alteration, c.1561G>T was confirmed to be a phenotype of the mild form, which was consistent with the study by Lualdi et al. [33].

Two silent mutations were identified in this study, c.684A>G (p.Pro228 =) and c.1122C>T (p.Gly374 =). The case with the nucleotide alteration, c.684A>G (p.Pro228 =), was referred from the NBS program and was confirmed to be a novel mutation and to be one of the combinations of the *IDS* mutations mentioned above. Another mutation, c.1122C>T (p.Gly374 =), has been reported to be pathogenic for the attenuated phenotype [20].

Six splicing mutations including c.103 + 34_56dup, c.240 + 1G>C, c.708 + 2T>G, c.880-2A>T, c.1006 + 5G>C, and c.1180 + 184T>C were identified, of which three were novel *IDS* mutation genes and the other three were pathogenic for either the severe form or the attenuated form of MPS II. As mentioned above, the variation allele, c.103 + 34_56dup, a novel *IDS* variation located between exon 1 and exon 2 (intron-1) downstream of the 34 to 56 regions, had a repeat sequence, CCTTCCTCCCTCCCTTCCTTCCT. The cDNA sequencing analysis was normal, and there was no significant difference in RNA expression in real-time PCR analysis [41]. Most of the cases also showed other *IDS* mutations, including c.851C>T (P284L) in exon 6, c.1180 + 184T>C (splicing) in intron 8, and c.684A>G (p.Pro228 =) in exon 5 (silent mutation). The patients with these combinations of variation alleles had pseudo-deficiencies of leukocyte IDS enzyme activities, ranging from 0.56 to 14.69 μmol/g protein/4 h, but no significant signs or symptoms were noted when surveying the family members who had the same variation alleles [41]. Chang et al. reported that the c.240 + 1G>C variation in intron 2 resulted in false splicing that caused the deletion of 105 amino acids and caused the severe form of MPS II [14]. The splicing mutation, c.708 + 2T>G has been reported to lead to the loss of a splice site that introduces 12 new AA then termination, with the base change of Aggt → AGGG [22]. In general, a severe phenotype was confirmed. Birot et al. reported that another splicing mutation, c.1006 + 5G>C, would lead to splicing in 22 nucleotides in intron 7 and cause the attenuated form of MPS II [11].

Four small deletions were found in our study, including c.231_236delCTTTGC, c.1055del12, c.1184delG, and c.1421delAG, all of which have been reported previously [17]. Mutation c.231del6 resulted in the loss of F78 and A79 and led to the severe form of MPS II according to clinical judgment. Mutation c.1055del12 caused a 12 bp deletion that caused protein alterations including the loss of V353-H356 and a severe phenotype. Mutation c.1184delG caused a 1 bp deletion resulting in the alteration of 44 amino acids by frame shift to stop codon, and a severe phenotype was observed. Mutation c.1421delAG caused a 2 bp deletion that led to the alteration of 17 amino acids by frame shift to termination, and a severe phenotype was also observed. The severe forms caused by these small deletions all corresponded well with the clinical symptoms and manifestations of the MPS II patients in our hospital.

**Figure 2:**
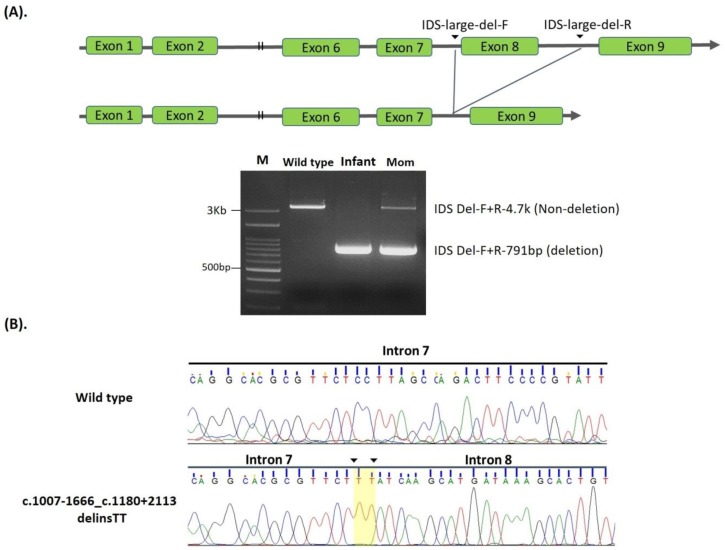
Scheme for generating a delins c.1007-1666_c.1180 + 2113delinsTT between intron 7 and intron 8. (**A**): Scheme for generating a delins c.1007-1666_c.1180 + 2113delinsTT between intron 7 and intron 8, leading to the loss of exon 8 in open reading frames. PCR using a primer pair flanking the delins site was used to detect this mutation in one infant and the carrier mother. Lanes M, wild-type, infant, and mom, corresponding to DNA size markers, wild type control, infant, and the mother. (**B**): Electropherogram showing the sequences of PCR products flanking the breakpoint created in this delins; note a dinucleotide TT insertion is shadowed.

Three gross deletions, exon 4–7 deletion, c.1007-1666_c.1180 + 2113delinsTT, and exon 8 deletions were found and were associated with the attenuated form of MPS II [11], except for one infant with an exon 8 deletion who was referred from the NBS program with no MPS pre-symptoms. A sequence of exon 8 linking c.1007-1666 and c.1180 + 2113, a total of 3953 bp, had been completely deleted, and TT insertion was observed in the deleted region between intron 7 and 8 (Figure 2A,B).

**Figure 3:**
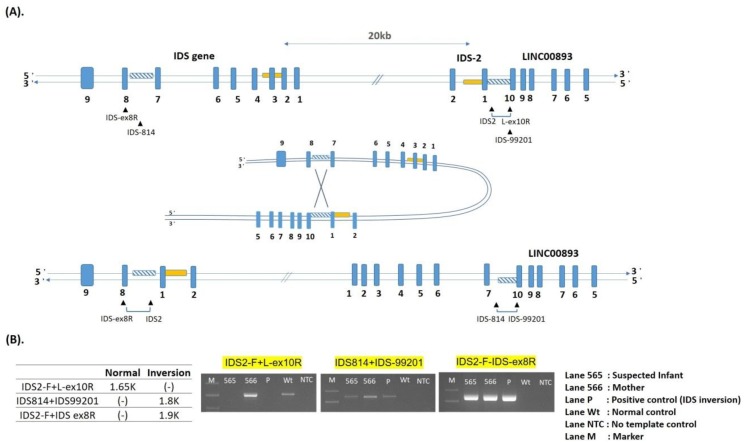
Scheme for generating a complex inversion in the *IDS* gene and *IDS-2* pseudogene. The created inversions were identified by PCR. (**A**): The supposed folding structure was formed by homologous sequences (twill line boxes) in the *IDS* gene and *IDS-2* pseudogene. The rearrangement of the *IDS* gene was inverted from the homolog in intron 7 to the homolog in distal *IDS-2*-LINC00893 region. (**B**): Three sets of primer pairs, as illustrated in Figure A that flanked the breakpoints of recombination were used; the lengths of the amplified fragments of created inversions were 1.9 kb in *IDS-2*, and 1.8 kb in *IDS*. PCR products of the affected infant and mother were resolved by electrophoresis, lanes 565, 566, P, Wt, NTC, and M, corresponding to the infant, mother, positive control, wild-type control, template control, and 100 bp ladder DNA size marker.

One complex rearrangement, *IDS* inversion, was identified. Recombination between the *IDS* gene and its putative pseudogene, *IDS-2*, resulted in an inversion of the intervening DNA. This inversion, which may have been the consequence of an intra-chromosomal mispairing, was caused by homologous recombination between sequences located in intron 7 of the *IDS* gene and sequences located distal of exon 3 in *IDS-2* [38,39]. *IDS* inversion led to the attenuated form of MPS II (Figure 3A,B).

### 2.2. The IDS Activity in Extracts of COS-7 Cells Expressing Novel Mutant cDNA

**Figure 4:**
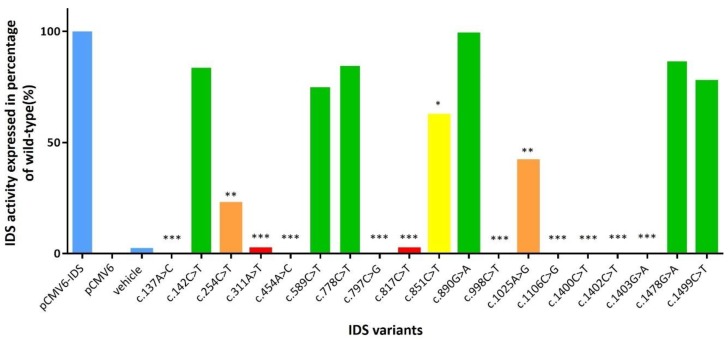
The percentages of IDS activity expressed in transfected COS-7 cells of individual novel missense mutations (*n* = 17), and previously reported missense mutation (*n* = 2). A *p*-value < 0.05 was considered to be statistically significant (*); *p*-value < 0.01 was considered to be highly significant (**); and a *p*-value < 0.001 was considered to be extremely significant (***).

A total of 19 mutant cDNA expression clones were constructed and analyzed for IDS activity in extracts of COS-7 cells in order to verify the influence of individual *IDS* variants found in the MPS II patients with different severities (Table S1). From the 19 mutants, 11 were novel mutants, which have not been reported and analyzed for enzyme activity in COS-7 cells, i.e., c.142C>T, c.254C>T, c.311A>T, c.454A>C, c.589C>T, c.778C>T, c.817C>T, c.890G>A, c.1025A>G, c.1478G>C and c.1499C>T. Six mutants have been reported previously but the *IDS* in vitro study has not been performed, i.e., c.137A>C, c.797C>G, c.851C>T, c.998C>T, c.1106C>G, and c.1400C>T. The rest two mutants, i.e., c.1402C>T and c.1403G>A, have been reported previously [17,21,29,30]. The IDS activities in wild-type, PCMV6-vector, vehicle, COS-7 cells (mock), and positive controls (c.928C>T, p.Q310Ter) were 768.49 (± 72.92), 59.66 (± 11.84), 91.80 (± 14.30), 72.99 (± 17.28), and 53.83 (± 10.79) μmol/g protein/4 h, respectively. The percentages of IDS activity expressed in transfected COS-7 cells of individual novel missense mutations for c.137A>C, c.311A>T, c.454A>C, c.797C>G, c.817C>T, c.998C>T, c.1106C>G, c.1400C>T, c.1402C>T, and c.1403G>A ranged from 0% to 2.2%, and were extremely significant compared to the wild type (*p* <0.001). In addition, the IDS activity expressions were 22.6% and 41.8% for c.254C>T and c.1025A>G mutations, respectively, which were highly significant compared to the wild type (*p* < 0.01), and the IDS activity expression of c.851C>T was 62.3% which was significant compared to the wild type (*p* < 0.05). The percentages of IDS activity expressed in transfected COS-7 cells of the other missense mutations, i.e., c.142C>T, c.589C>T, c.778C>T, c.890G>A, c.1478G>C, and c.1499C>T were 83.6%, 74.9%, 84.5%, 98.9%, 86.5%, and 77.5%, respectively, and there were no significant differences. The variations between repetitions of the enzyme activity tests were valid and acceptable, and the IDS values were the average of three different transfections (Figure 4). The results corresponded well with the quantities of uGAG-derived disaccharides and the IDS activities in leukocytes (Table 2). In Table 2, the missense mutations, i.e., c.254C>T, c.311A>T, c.817C>T, c.1025A>G, and c.1400C>T showed “positive” uGAG test results (DS: 11.59–45.95 μg/mL and HS: 11.43–30.01 μg/mL) and a deficiency in IDS enzyme activity (0.20–0.83 μmol/g protein/4 h) in leukocytes, which agrees with the disease prediction of the severe phenotype. In contrast, the infants with the other novel missense mutations, i.e., c.142C>T, c.589C>T, c.778C>T, c.890G>A, c.1478G>A, c.1499C>T, and c.1513T>C, showed “negative” uGAG test results and reductions in leukocyte IDS activities, indicating that the phenotype of these mutation variants could cause the attenuated form of MPS II disease. Intensive long-term follow-up examinations for asymptomatic MPS II infants are very important. In addition to the severe form found in the patients with missense mutations, six confirmed MPS II patients with the mutations c.253G>A, c.683C>T, c.697A>G, c.797C>G, c.801G>T, and c.1600A>C were diagnosed and confirmed to have the attenuated phenotype characterized by somatic problems, including developmental delays, stiffness of the joints, short stature, coarse facial features, especially the lips, nostrils, and tongue, enlargement of the head, progressive hearing loss, broad chest, and short neck, but without neuronopathic findings [17,18,22,32].

### 2.3. The Quantitative Analysis of Urinary GAG-Derived Disaccharides by Liquid Chromatography /Tandem Mass Spectrometry Assay

The values of DS and HS of the confirmed MPS II patients and the suspected MPS II infants calculated using the CS-normalized method varied widely, from 0.01 to 45.95 μg/mL for DS and from 0.09 to 203.35 μg/mL for HS. The relationships between uGAG-derived disaccharide levels, including DS and HS and the severity of the phenotype, were investigated, and the values of DS and HS closely matched the phenotypes of MPS. The MPS II patients with mental retardation had significantly higher levels of HS than those without mental retardation, and the DS values in the MPS II patients with hernia, hepatosplenomegaly, claw hands, coarse face, valvular heart disease, and joint stiffness were higher than in those without the symptoms reported by Lin et al. [42]. In the current study, “positive” uGAG tests indicated that the Dimethylmethylene Blue/creatinine (DMB/Cre.) ratio was increased, and distinct patterns of DS and HS separations were found on cellulose acetate sheet by two-dimensional electrophoresis (2-DEP), and the quantities of urinary DS and HS were significantly elevated in the MS/MS-based method. The confirmed MPS II patients and the seven highly suspected infants closely matched the diagnostic criteria of the MPS II phenotype.

## 3. Discussion

The relationship between the phenotype and genotype of MPS type II has been widely discussed, and the issue is of particular importance since the initiation of the NBS program for MPS in Taiwan. Almost none of the suspected MPS infants in this study had MPS pre-symptoms, although the first-line biochemistry examinations, IDS enzymatic assays, and *IDS* mutation gene analyses exhibited positive results. Infants in Taiwan are not permitted to receive ERT for Hunter syndrome unless their clinical and laboratory findings meet the guidelines for treatment issued by the National Health Insurance Bureau of Taiwan, which include the onset of one or more MPS signs or symptoms, deficiency of leukocyte IDS activity, identification of *IDS* variants, elevated urinary GAG levels, and definite evidence of a family history. The nationwide NBS program for MPS was officially launched in 1 August 2015, and up to 31 July 2019, a total of 324,426 infants had been screened in two newborn screening centers of Taiwan (Chinese Foundation of Health; CFOH and Taipei Institute of Pathology; TIP). In those, a total of 154 suspected infants who were referred to MacKay Memorial Hospital for confirmation due to the lower IDS enzyme activities in dried blood spot (DBS), which was measured by tandem mass spectrometry in the first test and the second recall test. The cut-off values of the first and the second test were < 6.5 and < 2.2 μmol/L/h, respectively. Genotyping was also performed when the second newborn screening specimen again had decreased enzyme activity [37]. In this study, seven infants were confirmed as MPS II based on the results of confirmatory diagnosis including a “positive” in uGAG biochemistry examinations, deficiency in leukocyte IDS enzyme activity, and identification of *IDS* variant. Of these seven infants, two were enrolled in ERT Program for MPS II; one received ERT (Elaprase; Shire Human Genetic Therapies, Boston, MA, USA) and another received ERT plus HSCT at the age of 1.11 and 0.55 years, respectively, due to definite evidence of family history. In order to achieve the goal of the NBS program for MPS, namely “Early detection, making an early diagnosis, giving them early therapy and preventing the onset of irreversible symptoms”, a comprehensive understanding of genotype–phenotype correlations is important by analyzing the genomic findings via in vitro expression studies. In this study, we evaluated the genotype–phenotype relationships of all 51 *IDS* variants currently known in Taiwan by integrating clinical data with in vitro expression studies and mass spectrometry-based assays of uGAGs to investigate and predict the likelihood of novel *IDS* variants, which can cause the severe or attenuated phenotype.

A total of 191 cases were enrolled in this study, including 51 confirmed MPS II patients (aged from 3.5 to 48.3 years) and 140 suspected MPS II infants who were referred from the NBS program for MPS. Of these cases, 66 had missense mutations, 3 had nonsense mutations, 4 had silent mutations, 106 had splicing mutations, 5 had small deletions, 3 had gross deletions, and 4 had complex rearrangements (*IDS* inversion). Twelve missense mutations including c.137A>C, c.189T>G, c.262C>T, c.413A>G, c.454A>C, c.998C>T, c.1039A>G, c.1402C>T, c.1403G>A, c.1454T>G, c.1466G>A, and c.1478G>C have been reported to cause the severe phenotype [13,14,16,17,18,22,23,26,27,28,29] according to clinical manifestations of neuronopathic with intellectual disabilities and cognitive decline with hyperactive and aggressive behavior, as well as in vitro expression studies [23,43]. Of these mutations, only one missense mutation, c.454A>C, was a novel variant that showed 0.0% IDS activity of the wild type (extremely significance, *p* < 0.001). Of the missense mutations, all of the novel variants except c.454A>C were found in infants from the NBS program and showed varied IDS activities in the extracts of COS-7 cells, i.e., c.142C>T (83.6%), c.254C>T (22.6%), c.311A>T (2.2%), c.589C>T (74.9%), c.778C>T (84.5%), c.817C>T (2.2%), c.890G>A (98.9%), c.1025A>G (41.8%), c.1478G>A (86.5%), c.1499C>T (77.5%), and c.1513T>C (not showed in this report) (Figure 4).

**Figure 5:**
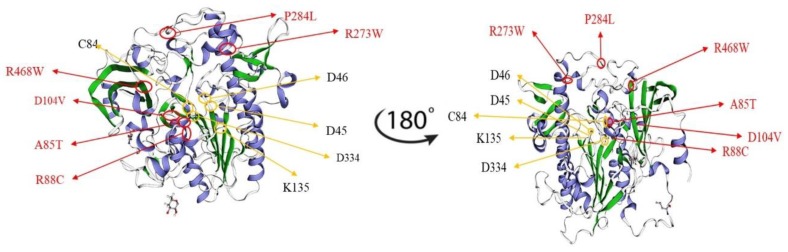
3D structure analysis by simulating six missense residues, i.e., c.253G>A (p.A85T), c.262C>T (p.R88C), c.311A>T (p.D104V), c.817C>T (p.R273W), c.851C>T (p.P284L), and c.1402C>T (p.R468W), was performed using SWISS-MODEL. The location and the residues of A85T and P284L may have had less influence on the structure of IDS protein and its function and were considered to cause the attenuated phenotype. The altered residues that may have strongly influenced the confirmation of the active site on IDS protein were identified by 3D structure analysis, i.e., R88C, D104V, R273W, and D104V variants may have been pathogenic variations for the severe phenotype of Hunter syndrome.

In order to investigate and assess the consequences of *IDS* missense variants on clinical phenotypes, the tertiary structure of *IDS* was constructed using the arylsulfatase structure as a template in homology modeling analysis. Structural analysis indicated that the residues of the mutations found in the severe phenotype had direct interactions with the active site residues or broke the hydrophobic core domain of *IDS*, whereas residues of the missense mutations found in the attenuated phenotype were located in the peripheral region as reported by Kato et al. [6]. The putative active site residues i.e., D45 (c.133-135GAT), D46 (c.136-138GAC), C84 (c.250-252TGC), K135 (c.403-405AAA), and D334 (c.1000-1002GAT) would lead to a severe phenotype of Hunter syndrome. In the current study, a 3D structural analysis was performed by simulating 6 missense residues, i.e., c.253G>A (p.A85T), c.262C>T (p.R88C), c.311A>T (p.D104V), c.817C>T (p.R273W), c.851C>T (p.P284L), and c.1402C>T (p.R468W) in the automated protein structure homology-modeling server, SWISS-MODEL (https://swissmodel.expasy.org) (Figure 5). Figure 5 shows that P284L residue on the loop (random coil) located at the peripheral region of the IDS structure that was far from the active site and was considered to be less conserved residue, and thus the variant would not have a strong influence on IDS protein configuration and function. R273W residue on the folding α-helix located between the peripheral region and active site of IDS structure was considered to be a conserved residue that would affect IDS protein function. According to our in vitro expression study, variants of the *IDS* gene would conserve about 2.2% of IDS activity of the wild type. R468 was adjacent to the positively charged active site residue. According to Kato et al., R468L or R468W, that is, the changes from the positively charged residue, Arginine, to large hydrophobic residues, Leucine or Tryptophan, should result in modification of the active site geometry and also in a significant change in substrate affinity [6]. R88 mutations were related to the severe phenotype. According to the structural analysis reported by Kato et al., the R88C residue itself was one of the most important active site residues. This finding suggests that any type of amino acid substitution would affect enzymatic activity in the severe phenotype, as observed in our patients [6,18,27,44,45]. In addition, there was no clear phenotype/genotype relationship associated with the A85T mutation; however, the location of this variant residue was adjacent to the active site domain and the changes in the non-polar amino acid, Alanine, to the polar amino acid with hydroxyl group, Threonine, which was extremely non-conserved and may result in the severe form of Hunter syndrome [6,27,46].

Interestingly, a complex rearrangement (*IDS* inversion) was found in four unrelated patients, in whom one case was referred from the NBS program for MPS. The pseudogene (*I2S2/IDSP1*) located on the telomeric side of *IDS* has been shown to undergo homologous recombination leading to large complex genomic/genetic rearrangements, which comprise about 13% of mutations [10,36]. Patients with this mutation had the attenuated phenotype. The region of *IDS* gene was inverted as a recombination from intron 7 to the distal part of exon 3 in *IDS-2*. The *IDS* inversion was not found after sequencing all nine exons of the *IDS* gene and their intro-exon junctions using conventional PCR-based method [47]. However, when using three pairs of designed primers, i.e., *IDS2-F + L-ex10R*, *IDS814 + IDS-99201*, and *IDS2-F + IDS-ex8R*, as indicated in Figure 3A for PCR-based analysis that flanked the breaking points of these two inversions, the inverse mutations were easily resolved. The length of PCR products in the suspected infant (Figure 3B, lane 565) and the mother (Figure 3B, lane 566) were 1.8 kb in *IDS* inversion and 1.9 kb in *IDS2* inversion, respectively. These PCR fragments were not detected from the wild *IDS* loci, however, the primer pair (*IDS2-F + ex10R*), which crossed the homolog of *IDS2* and LINC00893 junction resulted in a 1.65-kb PCR product (Figure 3B, lane Wt) [38,39]. In this study, the mother (lane 566) was confirmed to be a carrier and was positive in all PCRs.

One suspected infant had the c.1007-1666_c.1180 + 2113delinsTT mutation. In this case, a 3953 bp deletion and a dinucleotide TT insertion, which removed all exon 8 linking between c.1007-1666 and c.1180 + 2113 were observed (Figure 2A). The primers located in intron 7 and 8, respectively, successfully detected the large deletion, and the sequences of the PCR products showed the breaking point and TT insertion (Figure 2B). The phenotype of this infant with the gross deletion was indefinite, and further studies to predict the disease-causing potential of this novel variant are necessary to confirm the clinical significance and determine the effect of the variation on IDS protein function.

Five of the patients had small deletions (i.e., c.231_236delCTTTGC, c.1055del12, c.1184delG, and c.1421delAG) and they had the severe form phenotype. The patients were unrelated except for a male sibling with c.1421delAG. In general, small deletions of the *IDS* gene led to the loss of amino acids and the occurrence of frame shift to termination when in translation a stop codon was interpreted that may have altered the normal arrangement or sequence of amino acids in the *IDS* gene, resulting in serious defects of IDS protein structure and function.

A total of 99 suspected infants were identified with the variation allele, c.103  +  34_56dup, which was linked with other variants, including c.851C>T (P284L) in exon 6, c.1180 + 184T>C (splicing) in intron 8, and c.684A>G (p.Pro228 =) in exon 5 (silent mutation). For those cases, all the results showed “negative” in urinary first-line biochemistry examinations and “reduction” of leukocyte IDS activities varied from 0.56 to 14.69 μmol/g protein/4 h, which did not exactly meet the confirmatory criteria for a typical MPS II diagnosis. According to our cDNA sequencing and RNA real-time PCR analyses, the combinative variation alleles, c.103  +  34_56dup plus c.1180 + 184T>C, showed normal in cDNA sequences and no significant difference of mRNA levels; besides, the silent mutation, c.684A>G (p.Pro228 =) in exon 5, that would not cause the change of the amino acid Proline that was defined as non-pathogenic, not leading to the defect of IDS protein. In addition, the only variation that would cause pseudo-deficiency of IDS enzyme activity was c.851C>T (P284L), in which the in vitro expression of IDS activity was 62.3% of the wild type. The single nucleotide change, and the deduced amino acid substitution might produce a steric effect that mildly influences the stability of mRNA and/or folding of IDS protein. In summary, the frequency of the Taiwanese population carried with the combination of above four variants was high, about 70.7% of referred cases (99/140), and the infants with these combined variations might not show up any notable MPS presentations, but it is still required for a long-term follow-up inspection of disease progression, even though the male family members with the same variations via maternal inheritance have been thoroughly investigated. In our study, an additional two suspected cases were notable; one had the c.103  +  34_56dup variation accompanied with c.851C>T (P284L), and another had the variation allele c.851C>T plus c.1180  +  184 T>C. Variation c.851C>T (P284L) was reported by Sawada et al. that this substitution might cause pseudo-deficiency of IDS and might result in a structural modeling of the enzyme (http://www.aismme.org/pdf/ACIMD.pdf). From the report, *IDS* with c.851C > T (P284L) confirmed that this amino acid substitution was non-pathogenic. In addition, another study reported that the missense mutation c.851C > T (P284L) could cause an attenuated phenotype of Hunter syndrome according to the exhibition of some clinical presentations [24].

## 4. Materials and Methods

### 4.1. Patients and Samples

A total of 191 cases were enrolled and analyzed in this study. These cases included 51 patients diagnosed with MPS II since the 1990s in Taiwan, and 140 infants suspected of having MPS II after being screened by the Newborn Screening (NBS) Program for MPS. This screening program was established in Taiwan in August 2015, and all children suspected of having MPS were then referred to MacKay Memorial Hospital to confirm the diagnosis. The samples required for the assays included urine (10–20 mL) and EDTA blood (2 tubes, 3–5 mL in each). Urine samples were stored at −20 °C prior to GAG analyses, and the blood samples were kept at room temperature and 4 °C before leukocyte isolation for enzymatic assay and molecular DNA analysis, respectively. All procedures were performed in accordance with the ethical standards of the responsible committees on human experimentation (institutional and national) and with the Declaration of Helsinki of 1975, as revised in 2000. The Institutional Review Board of MacKay Memorial Hospital approved this study (14MMHIS281, approval date: 30 March 2015; 16MMHIS152, approval date: 22 June 2017; and 17MMHIS176, approval date: 30 August 2018), and written informed consent was obtained from all of the patients or their parents.

### 4.2. DNA Isolation, Amplification, and Sequencing

Genomic DNA was extracted using a QIAamp DNA Blood Mini Kit (Qiagen, Hilden, Germany) according to the manufacturer’s instructions, from peripheral leukocytes of all patients. All *IDS* gene exons and flanking intronic regions were amplified by PCR using Taq DNA polymerase 2X Master Mix Red (containing Tris-HCl pH 8.5, (NH_4_)_2_SO_4_, 3 mM MgCl_2_, 0.2% Tween 20, 0.4 mM dNTPs, 0.2 units/µL Ampliqon Taq DNA polymerase, inert red dye and stabilizer; Ampliqon, Denmark), 10 pmol of each forward and reverse primer, and 75 ng of template DNA in a final volume of 30 µL. The PCR conditions were as follows: 94 °C for 5 min, 33 cycles of 94 °C for 40 s, 58–62 °C for 40 s and 72 °C for 40 s, and a final extension time of 72 °C for 10 min. The PCR products were analyzed by 2.5% agarose gel electrophoresis, stained with SYBER green, and then visualized under a UV trans-illuminator. Sequencing of PCR products was performed at Genomics Inc. sequencing services (New Taipei City, Taiwan) on an ABI 3730XL DNA analyzer (Applied Biosystems).

### 4.3. Constructing Mutant DNA by Site-Directed Mutagenesis

Plasmids expressing IDS protein were made by cloning the *IDS* cDNA into a pCMV6-Entry based Myc-DDK tagging vector (ORIGENE, RC219187) (Figure S1). Nineteen mutations of the *IDS* gene, including c.137C>A (p.D46A), c.142C>T (p.R48C), c.254C>T (p.A85V), c.311A>T (p.D104V), c.454A>C (p.S152R), c.589C>T (p.P197S), c.778C>T (p.P260S), c.797C>G (p.P266R), c.817C>T (p.R273W), c.851C>T (p.P284L), c.890G>A (p.R297H), c.998C>T (p.S333L), c.1106C>G (p.S369X), c.1025A>G (p.H342R), c.1400C>T (p.P467L), c.1402C>T (p.R468W), c.1403G>A (p.R468Q), c.1478G>A (p.R493H), and c.1499C>T (p.T500I) were introduced into the wild-type *IDS* cDNA by site-directed mutagenesis. Table S2 lists the primers used for site-directed mutagenesis. PCR was carried out with initial denaturation at 95 °C for 2 min, 18 cycles of denaturation at 95 °C for 20 s, annealing at 60 °C for 10 s, and extension at 68˚C for 30 s followed by a final extension for 5 min. The reaction mixture contained 10 ng DNA template, 125 ng of oligonucleotide primers, 5 µL of 10X Reaction Buffer, 1 µL of dNTP, 1.5 µL of QuikSolution reagent and 1 µl of QuikChange Lightning Enzyme (QuikChange Lightning Site-Directed Mutagenesis Kit, Agilent Technologies, Santa Clara, CA, USA). The constructs were verified by direct DNA sequencing.

### 4.4. Cell Culture and Transient Transfection

COS-7 cells were cultured in Dulbecco’s MEM (Gibco; Thermo Fisher Scientific, Grand Island, NJ, USA) supplemented with 10% fetal bovine serum (Gibco; Thermo Fisher Scientific, Grand Island, NJ, USA) and penicillin-streptomycin mixture (Gibco; Thermo Fisher Scientific, Grand Island, NJ, USA) at 37 °C in 5% CO_2_. The COS-7 cells were transfected by pCMV6-Entry plasmids of wild-type *IDS* cDNA and the 19 mutants using Lipofectamine 3000 (Invitrogen, Carlsbad, CA, USA) following the manufacture’s protocol. After 24 h of incubation at 37 °C, the cells were harvested for IDS enzyme assays. These experiments were performed in triplicate.

### 4.5. Enzyme Assay for MPS II

Leukocyte enzyme assay for MPS II was described previously [40,48]. The enzymatic liberation of fluorochrome from 4MU-α-L-iduronide-2-sulfate required the sequential action of IDS and α-iduronidase. The rate of fluorescence increase was directly proportional to enzyme activity. Leukocyte isolation and protein determination were required prior to performing an enzyme assay. Leukocytes were isolated from EDTA blood by centrifugation through Ficoll-Paque (Sigma-Aldrich, Inc., St. Louis, MO, USA) at 18 °C for 40 min at 2500 rpm. By removing the upper layer, the white cell ring from the interface was removed and transferred to a 5 mL centrifuge tube, followed by the addition of 0.9% NaCl to the top, mixing, and centrifugation for 10 min at 2000 rpm at 4 °C. Cell lysates were prepared by suspending leukocyte pellets in 0.2 mL of 0.85% NaCl and disrupted by 6 cycles of freeze-thawing. Proteins were determined using Coomassie Plus protein assay (Pierce, Thermo Fisher Scientific Inc., Waltham, MA, USA). The assay for individual enzyme activity was performed using 4-methylumbelliferyl substrate, with the enzyme activity being proportional to the amount of liberated fluorescence detected (μmol enzyme activity/g protein/hour). Individual enzyme activity, which was 5% lower than normal was defined as a marked reduction in that enzyme activity. The principle of the assay has been illustrated previously [41,48,49]. The enzymatic liberation of fluorochrome from 4MU-α-L-iduronide-2-sulfate required the sequential action of IDS and α-iduronidase, and a normal level of α-iduronidase activity was insufficient to complete hydrolysis of the reaction intermediate 4-methylumbelliferyl-α-iduronide formed by IDS. In performing IDS enzymatic assay, at least one additional sulfatase is recommended to analyze to differentiate from multiple sulfatase deficiency. In our study, one additional enzyme, i.e., α-L-iduronidase (IDUA), was analyzed by 4-MU fluorometric assay to ensure the quality assurance of the cell supernatant sample.

### 4.6. The Quantification of uGAG-Derived Disaccharides by Tandem Mass Spectrometry Assay

The Tandem mass spectrometry (liquid chromatography/tandem mass spectrometry; LC-MS/MS) (4000 QTRAP LC-MS/MS System; AB Sciex, Foster City, CA, USA) assay for relevant GAG-derived disaccharides was performed using methanolysis for chondroitin sulfate (CS), DS and HS [50,51,52], and the mass to charge(m/z) of the parent ion and its daughter ion after collision was 426.1→236.2 for DS and 384.2→161.9 for HS. The quantities of urinary DS and HS were determined by applying the CS-normalized method reported by Lin et al., and the reference cut-off values were < 0.80 μg/mL for DS and < 0.78 μg/mL for HS [53].

### 4.7. MPS II Phenotype Determination

The clinical signs and symptoms of MPS II were heterogeneous, and the disorder should be regarded as two broad groups, the severe and attenuated forms, according to the severity of symptoms and whether or not the patients are neuronopathic with intellectual disability. Neuronopathic with intellectual disability was assessed according to the scores for IQ and DQ tests and the Functional Independence Measure for Children (WeeFIM) questionnaire for the parents [54]. Individuals with attenuated MPS II were most often diagnosed between the ages of 4 and 8 years, and survival to adulthood was common. Patients with the more severe form of MPS II exhibited a chronic and progressive disease involving multiple organs and tissues. The age at diagnosis was usually between 18 and 36 months, and death from a combination of neurological deterioration and cardio-respiratory failure usually occurred in the mid-teenage years [14]. Patients who underwent an HSCT or ERT could prolong their life and dramatically change the natural history of this disease. For this reason, it was mandatory to have a correlation between the severity of MPS II and genotype to decide the appropriate treatment.

### 4.8. Statistical Analysis

Statistical analysis (Students t-test) was performed to compare values of IDS activity obtained from individual variants to those of the wild type. *P* values were calculated using Excel 2010 (Microsoft Office), and a *p*-value < 0.05 was considered to be statistically significant, a *p*-value < 0.01 was considered to be highly significant, and *p*-value < 0.001 was considered to be extremely significant.

## 5. Conclusions

Awareness of the specific mutations involved in MPS II may help to clarify the relationship between genotype and phenotype in individual patients and allow for the identification of female carriers. To characterize the biochemical and molecular defects in IDS-deficient patients and their families, this study was designed to identify *IDS* gene mutations in a group of Taiwanese patients with MPS II. Sixteen novel variants were found, and these cases were all referred from the NBS program for MPS in the past 4 years. Because such patients were too young to manifest signs or symptoms, the investigation of genotype–phenotype relationships was especially important. In this study, clinical data were integrated with in vitro expression studies as well as a mass spectrometry-based assay of uGAGs to predict the likelihood of novel *IDS* variants, which can cause the severe or attenuated phenotype. Further molecular investigations with a reliable intelligence or developmental disability score should be performed to better understand the genotype–phenotype correlation for future therapeutic approaches.

## Figures and Tables

**Table 1:** Mutations of the iduronate-2-sulfatase (*IDS*) gene underlying Taiwanese Hunter syndrome by sequencing analysis.

No.	Missense Nucleotide Alteration	Protein Alteration	Gene Location	Phenotype Severity	IDS Activity	uGAG Tests	Known/ Novel	ACMG Classification
1	c.137A>C	p.D46A	Exon 2	S	0.1	Positive	Known [16]	
2	c.142C>T	p.R48C	Exon 2	^#^NBS	16.27	Negative	Novel	Likely Pathogenic
3	c.189T>G	p.N63K	Exon 2	S	0.21	Positive	Known [17]	
4	c.253 G>A	p.A85T	Exon 3	A	0.00	Positive	Known [17,18]	
5	c.254C>T	p.A85V	Exon 3	^#^NBS	0.83	Positive	Novel	Likely Pathogenic
6	c.262C>T	p.R88C	Exon 3	S	0.43	Positive	Known [17,19,20]	
7	c.301C>T	p.R101C	Exon 3	^#^NBS	15.4-40.8	Negative	Known [21]	Benign
8	c.311A>T	p.D104V	Exon 3	^#^NBS	0.32	Positive	Novel	Likely Pathogenic
9	c.413A>G	p.H138R	Exon 3	S	0.18	Positive	Known [17]	
10	c.454A>C	p.S152R	Exon 4	S	0.11	Positive	Novel	Likely Pathogenic
11	c.589C>T	p.P197S	Exon 5	^#^NBS	7.8	Negative	Novel	Likely Pathogenic
12	c.683C>T	p.P228L	Exon 5	A	0.56	Positive	Known [17,22]	
13	c.697A>G	p.R233G	Exon 5	A	0.71	Positive	Known [20]	
14	c.778C>T	p.P260S	Exon 6	^#^NBS	6.47	Negative	Novel	Likely Pathogenic
15	c.797C>G	p.P266R	Exon 6	A	1.96	Positive	Known [22]	
16	c.801 G>T	p.W267C	Exon 6	A	0.89	Positive	Known [17]	
17	c.817C>T	p.R273W	Exon 6	^#^NBS	0.2	Positive	Novel	Likely Pathogenic
18	c.851C>T	p.P284L	Exon 6	^#^NBS (A)	0.51	Negative	Known [24]	Uncertain Significance
19	c.890G>A	p.R297H	Exon 7	^#^NBS	9.2	Negative	Novel	Likely Pathogenic
20	c.998C>T	p.S333L	Exon 7	S	0.34	Positive	Known [25,26]	
21	c.1025A>G	p.H342R	Exon 8	^#^NBS	0.4	Positive	Novel	Likely Pathogenic
22	c.1039A>G	p.K347E	Exon 8	S	0.49	Positive	Known [17]	
23	c.1400C>T	p.P467L	Exon 9	^#^NBS	0.27	Positive	Known [27,28]	Likely Pathogenic
24	c.1402C>T	p.R468W	Exon 9	S	0.04	Positive	Known [17,29]	
25	c.1403G>A	p.R468Q	Exon 9	S	0.00	Positive	Known [17,21,30]	
26	c.1454T>G	p.I485R	Exon 9	S	0.16	Positive	Known [17,31]	
27	c.1466G>A	p.G489D	Exon 9	S	0.11	Positive	Known [17]	
28	c.1478G>A	p.R493H	Exon 9	^#^NBS	8.82–124.91	Negative	Novel	Likely Pathogenic
29	c.1478G>C	p.R493P	Exon 9	S	0.13	Positive	Known [16,28]	
30	c.1499C>T	p.T500I	Exon 9	^#^NBS	13.2–34.5	Negative	Novel	Benign
31	c.1513T>C	p.P505L	Exon 9	^#^NBS	5.93	Negative	Novel	Likely Pathogenic
32	c.1600A>C	p.N534H	Exon 9	A	1.09	Positive	Known [32]	
	**Nonsense**							
1	c.801G>A	p.W267X	Exon 6	S	0.15	Positive	Known [17]	
2	c.1106C>G	p.S369X	Exon 7	A	0.1	Positive	Known [33]	
3	c.1561G>T	p.E521X	Exon 9	S	0.24	Positive	Known [17,34]	
	**Silent**							
1	c.684A>G	p.Pro228 =	Exon 5	^#^NBS	NA	NA	Novel	Benign
2	c.1122 C>T	p.Gly374 =	Exon 8	A	0.34–7.1	Positive	Known [20]	
	**Splicing**							
1	c.103 + 34_56dup		Intron 1	^#^NBS	0.56–14.69	Negative	Novel	Uncertain Significance
2	c.240 + 1G>C	False splicing; deletion of 105 AAs	Intron 2	S	0.68	Positive	Known [17]	
3	c.708 + 2T>G	−	Intron 5	S	0.48	Positive	Known [22]	
4	c.880-2A>T	−	Intron 7	A	0.75	Positive	Novel	Pathogenic
5	c.1006 + 5G>C	Splicing in 22 nucleotide	Intron 7	A	0.05	Positive	Known [35]	
6	c.1180 + 184T>C	−	Intron 8	^#^NBS	NA	NA	Novel	
	**Small Deletions**							
1	c.231_236delCTTTGC	Loss of F78 and A79	Exon 2	S	0.12	Positive	Known [17]	
2	c.1055del12	Loss of V353-H356	Exon 8	S	0.25	Positive	Known [17]	
3	c.1184delG	Frame shift, 44 altered AAs, term	Exon 9	S	0.19	Positive	Known [17]	
4	c.1421delAG	Frame shift, 7 altered AAs, term	Exon 9	S	0.34	Positive	Known [17]	
	**Gross deletions**							
1	Exon 4–7 deletion	NA		A	0.3	Positive	Known [11]	
2.	c.1007-1666_c.1180 + 2113 delinsTT	NA		^#^NBS	0.99	Positive	Known [36,37]	Pathogenic
3	Exon 8 deletion	NA		A	0.64	Positive	Known [37]	
	**Complex Rearrangements**							
1	*IDS* inversion	NA		A	0.13–1.54	Positive	Known [38,39]	

A combination of four mutations, including c.103 + 34_56dup, c.851C>T; p.P284L, c.1180 + 184T>C, and c.684A>G; p.Pro228 =. ^#^ NBS is the abbreviation of newborn screening; S: Severe; A: Attenuated.

**Table 2:** Mutations of the iduronate-2-sulfatase (*IDS*) gene found in suspected MPS II infants referred from newborn screening program for MPS in Taiwan.

No.	Missense Nucleotide Alteration/Protein Alteration	Ages (Ms) of the Test	Ages (Yrs) at last Follow up	^(a)^Leukocyte IDS Activity	^(b)^uGAG Tests	DMB/Cre Ratio	uDS (μg/mL)	uHS (μg/mL)	ACMG Classification
1	c.142C>T; p.R48C	1.7	0.3	16.27	Negative	38.73	0.01	0.75	Likely Pathogenic
2	c.254C>T; p.A85V	4.6	0.5	0.83	Positive	78.58	11.59	12.36	Likely Pathogenic
3	c.301C>T; p.R101C	1.7	3.4	15.4–40.8	Negative	5.07	0.2	0.13	Benign
4	c.311A>T; p.D104V	0.9	0.3	0.32	Positive	44.6	45.95	11.43	Likely Pathogenic
5	c.589C>T; p.P197S	1.9	3	7.80	Negative	63.82	0.38	1.46	Likely Pathogenic
6	c.778C>T; p.P260S	2.3	1	6.47	Negative	12.29	0.12	0.1	Likely Pathogenic
7	c.817C>T; p.R273W	0.9	0.7	0.20	Positive	65.06	15.78	16.23	Likely Pathogenic
8	c.851C>T; p.P284L	1.8	1.3	0.51	Negative	34.22	0.03	0.08	Uncertain Significance
9	c.890G>A; p.R297H	3	0.5	9.20	Negative	69.67	0.08	0.04	Likely Pathogenic
10	c.1025A>G; p.H342R	1.6	0.3	0.40	Positive	70.90	21.21	12.06	Likely Pathogenic
11	c.1400C>T; p.P467L	1.9	0.7	0.27	Positive	153.16	21.4	30.01	Likely Pathogenic
12	c.1478G>A; p.R493H	1.6	3.2	26.37 ± 10.98	Negative	40.18	0.04	0.09	Likely Pathogenic
13	c.1499C>T; p.T500I	1.6	1.9	17.15 ± 3.69	Negative	26.11	0.1	0.27	Benign
14	c.1513T>C; p.P505L	1.4	0.5	5.93	Negative	32.15	0.08	0.11	Likely Pathogenic
	**Splicing**								
15	* c.103 + 34_56dup	1.1	3.8	3.86 ± 2.24	Negative	41.15	0.06	0.11	Uncertain Significance
16	c.1180 + 184T>C			NA	NA	NA	NA	NA	
	**Gross Deletions**								
17	c.1007-1666_c.1180 + 2113 delinsTT (including exon 8 del)	1.2	1.2	0.99	Positive	177.96	30.77	203.35	Pathogenic
	**Complex Rearrangement**								
18	*IDS* inversion	1.5	0.3	0.13	Positive	44.05	8.72	37.30	

^(^^a)^ IDS enzyme activity (Ref. 12.89 ~ 131.83 μmol/g protein/4 h); ^(b)^ GAG tests including GAG quantification (DMB/Cre ratio): Reference values 39.9 ± 13.1 mg/mmol creatinine (< 6 months), 44.6 ± 23.7 (under 2 years old), 15.3 ± 13.0 (2–17 years old), 5.2 ± 2.5 (18–42 years old); 2-dimensional electrophoresis: Showing DS and HS patterns rather than CS; and the quantitative analyses of GAG-derived disaccharides (DS and HS) using tandem mass spectrometry assay (MS/MS-based method), cut-off values: < 0.80 μg/mL for DS; < 0.78 μg/mL for HS. *A combination of four mutations, including c.103 + 34_56dup, c.851C>T; p.P284L, c.1180 + 184T>C, and c.684A>G; p.Pro228 = (*n* = 99).

## Supplementary Materials

Supplementary Materials can be found at https://www.mdpi.com/1422-0067/21/1/114/s1.
