# Supplementary material for: Identification and Functional Characterization of IDS Gene Mutations Underlying Taiwanese Hunter Syndrome (Mucopolysaccharidosis Type II)
Source: Int J Mol Sci. 2019 Dec 23;21(1):114. doi: 10.3390/ijms21010114 (PMC6982257; doi:10.3390/ijms21010114)
Supplement: Supplementary file 1 [file ijms-21-00114-s001.zip › Supplementary table 1 (IJMS-special issue).pdf]

Supplementary Table 1.

**Transfected vector**	**IDS activity** (µmole/g protein/ 4hr)	**Total** (percentage of wild-type)
Wild-type	641.0 ± 98.72	100.0
PCMV6-vector	0.0 ± 8.09	0.0
vehicle	12.1 ± 24.05	1.9
c.137A>C(p.D46A)	0.0 ± 8.56	0.0
c.142C>T (p.R48C)	535.6 ± 48.78	83.6
c.254C>T(p.A85V)	134.0 ± 38.07	22.6
c.311A>T(p.D104V)	14.2 ± 12.98	2.2
c.454A>C (p.S152R)	0.0 ± 9.02	0.0
c.589C>T(p.P197S)	480.0 ± 90.94	74.9
c.778C>T (p.P260S)	541.9 ± 51.5	84.5
c.797C>G (p.P266R)	0.0 ± 8.51	0.0
c.817C>T(p.R273W)	14.3 ± 15.21	2.2
c.851C>T(p.P284L)	399.1 ± 86.34	62.3
c.890G>A(p.R297H)	634.3 ± 88.00	98.9
c.998C>T (p.S333L)	0.0 ± 5.11	0.0
c.1025A>G (p.H342R)	268.1 ± 62.02	41.8
c.1106C>G(p.S369X)	0.2 ± 12.35	0.0
c.1400C>T (p.P467L)	0.0 ± 9.49	0.0
c.1402C>T (p.R468W)	0.0 ± 7.19	0.0
c.1403G>A (p.R468Q)	0.0 ± 8.63	0.0
c.1478G>A,(p.R493H)	554.2 ± 90.10	86.5
c.1499C>T(p.T500I)	496.7 ± 99.85	77.5
