# Supplementary material for: Identification and Functional Characterization of IDS Gene Mutations Underlying Taiwanese Hunter Syndrome (Mucopolysaccharidosis Type II)
Source: Int J Mol Sci. 2019 Dec 23;21(1):114. doi: 10.3390/ijms21010114 (PMC6982257; doi:10.3390/ijms21010114)
Supplement: Supplementary file 1 [file ijms-21-00114-s001.zip › Supplementary Table 2 (IJMS-special issue).pdf]

Supplementary Table 2.

Mutants	Forward primers(5’-3’)	Reverse primers(5’-3’)
c.137A>C	ATCATCGTGGATGCCCTGCGCCCCTCC	GGAGGGGCGCAGGGCATCCACGATGAT
c.142C>T	GTGGATGACCTGTGCCCCTCCCTGG	CCAGGGAGGGGCACAGGTCATCCAC
c.254C>T	AGCAGTGTGCGTCCCGAGCCGCG	CGCGGCTCGGGACGCACACTGCT
c.311A>T	CCACCCGCCTGTACGTCTTCAACTCCTACTG	CAGTAGGAGTTGAAGACGTACAGGCGGGTGG
c.454A>C	ACCATACCGATGATTCTCCGTATCGCTGGTCTTTTC	GAAAAGACCAGCGATACGGAGAATCATCGGTATGGT
c.589C>T	CCGAGGGCACCTTGTCTGACAAACAGAGC	GCTCTGTTTGTCAGACAAGGTGCCCTCGG
c.778C>T	GGTCCCTGATGGCCTATCCCCTGTGGC	GCCACAGGGGATAGGCCATCAGGGACC
c.797C>G	CTGTGGCCTACAACCGCTGGATGGACATCAG	CTGATGTCCATCCAGCGGTTGTAGGCCACAG
c.817C>T	GGATGGACATCAGGCAATGGGAAGACGTCCA	TGGACGTCTTCCCATTGCCTGATGTCCATCC
c.851C>T	CCTTAAACATCAGTGTGCTGTATGGTCCAATTCCTGT	ACAGGAATTGGACCATACAGCACACTGATGTTTAAGG
c.998C>T	AGCACCATCATTGCATTTACCTTGGATCATGGGTGG	CCACCCATGATCCAAGGTAAATGCAATGATGGTGCT
c.1106C>G	CTGGAAGGACGGCTTGACTTCCGGAGGC	GCCTCCGGAAGTCAAGCCGTCCTTCCAG
c.1400C>T	GCCTATAGCCAGTATCTCCGGCCTTCAGAC	GTCTGAAGGCCGGAGATACTGGCTATAGGC
c.1402C>T	CTATAGCCAGTATCCCTGGCCTTCAGACATCC	GGGATGTCTGAAGGCCAGGGATACTGGCTATAG
c.1403G>A	ATAGCCAGTATCCCCAGCCTTCAGACATCCC	GGGATGTCTGAAGGCTGGGGATACTGGCTAT
c.1478G>A	GATCATGGGCTATTCCATACACACCATAGACTATAGGTATA	TATACCTATAGTCTATGGTGTGTATGGAATAGCCCATGATC
c.1499C>T	ACGCACCATAGACTATAGGTATATTGTGTGGGTTGG	CCAACCCACACAATATACCTATAGTCTATGGTGCGT
