# Supplementary figures and images for: Identification and Functional Characterization of IDS Gene Mutations Underlying Taiwanese Hunter Syndrome (Mucopolysaccharidosis Type II)
Source: Int J Mol Sci. 2019 Dec 23;21(1):114. doi: 10.3390/ijms21010114 (PMC6982257; doi:10.3390/ijms21010114)

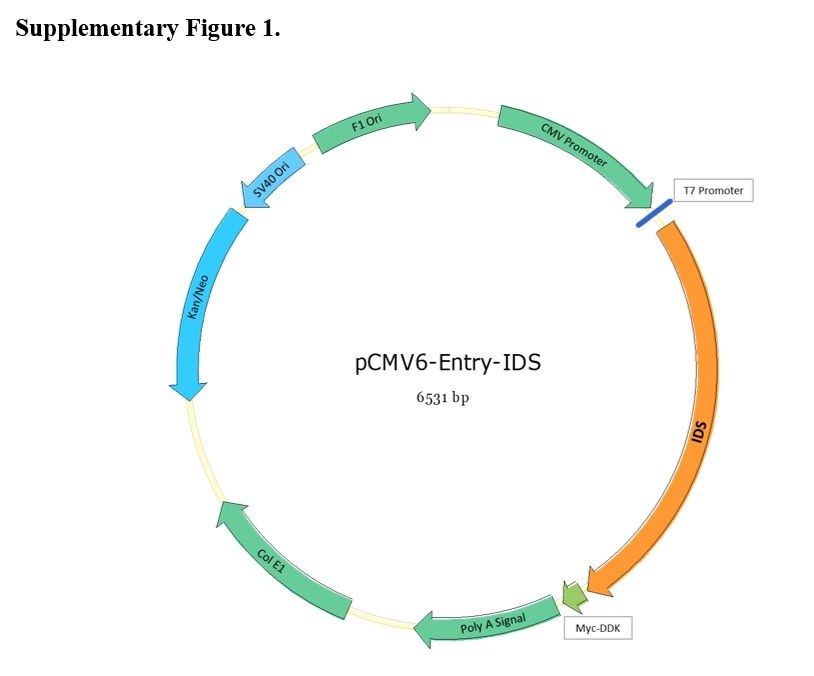

Supplement: Supplementary file 1 [file ijms-21-00114-s001.zip › supplementary figure 1(IJMS).jpg]
